# Prediagnostic CT or MRI Utilization and Outcomes in Hepatocellular Carcinoma: SEER-Medicare Database Analysis

**DOI:** 10.1158/2767-9764.CRC-23-0075

**Published:** 2023-05-16

**Authors:** Mohammad A. Karim, Amit G. Singal, Hye Chung Kum, Yi-Te Lee, Sulki Park, Nicole E. Rich, Mazen Noureddin, Ju Dong Yang

**Affiliations:** 1 Population Informatics Lab, School of Public Health, Texas A&M University, College Station, Texas.; 2 Division of Digestive and Liver Diseases, University of Texas Southwestern Medical Center, Dallas, Texas.; 3 California NanoSystems Institute, Crump Institute for Molecular Imaging, Department of Molecular and Medical Pharmacology, University of California, Los Angeles, Los Angeles, California.; 4 Karsh Division of Gastroenterology and Hepatology, Comprehensive Transplant Center, Cedars-Sinai Medical Center, Los Angeles, California.; 5 Samuel Oschin Comprehensive Cancer Institute, Cedars-Sinai Medical Center, Los Angeles, California.

## Abstract

Ultrasound-based surveillance has suboptimal sensitivity for early hepatocellular carcinoma (HCC) detection, generating interest in alternative surveillance modalities. We aim to investigate the association between prediagnostic CT or MRI and overall survival in a contemporary cohort of patients with HCC. Using the Surveillance Epidemiology and End Results (SEER)-Medicare database, we analyzed Medicare beneficiaries diagnosed with HCC between 2011 and 2015. Proportion of time covered (PTC) was defined as the proportion of the 36-month period prior to HCC diagnosis in which patients had received abdominal imaging (ultrasound, CT, MRI). Cox proportional hazards regression was used to investigate the association between PTC and overall survival. Among 5,098 patients with HCC, 3,293 (65%) patients had abdominal imaging prior to HCC diagnosis, of whom 67% had CT/MRI. Median PTC by any abdominal imaging was 5.6% [interquartile range (IQR): 0%–36%], with few patients having PTC >50%. Compared with no abdominal images, ultrasound [adjusted HR (aHR): 0.87, 95% confidence interval (CI): 0.79–0.95] and CT/MRI group (aHR: 0.68, 95% CI: 0.63–0.74) were associated with improved survival. Lead-time adjusted analysis showed improved survival continued to be observed with CT/MRI (aHR: 0.80, 95% CI: 0.74–0.87) but not ultrasound (aHR: 1.00, 95% CI: 0.91–1.10). Increased PTC was associated with improved survival, with a larger effect size observed with CT/MRI (aHR per 10%: 0.93, 95% CI: 0.91–0.95) than ultrasound (aHR per 10%: 0.96, 95% CI: 0.95–0.98). In conclusion, PTC by abdominal images was associated with improved survival in patients with HCC, with potential greater benefit using CT/MRI. Regular utilization of CT/MRI before cancer diagnosis may have potential survival benefit compared to ultrasound in patients with HCC.

**Significance:**

Our population-based study using SEER-Medicare database demonstrated that proportion of time covered by abdominal imaging was associated with improved survival in patients with HCC, with potential greater benefit using CT/MRI. The results suggest that CT/MRI surveillance may have potential survival benefit compared with ultrasound surveillance in high-risk patients for HCC. A larger prospective study should be conducted for external validation.

## Introduction

Patients with cirrhosis or chronic hepatitis B virus (HBV) infection have increased risk of hepatocellular carcinoma (HCC; ref. 1). Surveillance allows early-stage HCC detection when patients do not have cancer-related symptoms and is associated with a high chance of receiving potentially curative treatment (2). Therefore, major professional societies recommend HCC surveillance in patients with cirrhosis or high-risk HBV infection (3–5).

Ultrasound, the currently recommended surveillance test, has suboptimal sensitivity to detect HCC at an early stage (6). Furthermore, accuracy of ultrasound is decreased in patients with abdominal obesity, nonalcoholic fatty liver disease (NAFLD), and alcohol-associated cirrhosis—rapidly growing populations—due to increased echogenicity and heterogeneous echotexture, limiting the visualization of small liver nodules (7–9).

Because of the limitation of ultrasound, CT and MRI have been entertained as alternative surveillance tests (10). Multiple studies showed that CT and MRI have higher accuracy than ultrasound for early HCC detection (10–13), and decision analyses suggest that MRI-based surveillance test may be cost-effective in high-risk individuals with annual incidence rates exceeding 2%–3% (14–16). However, the American Association For the Study of Liver Diseases guidelines recommend against the use of CT or MRI as primary modalities for HCC surveillance in patients with cirrhosis and reserve their use in select patients with a history or high likelihood of inadequate ultrasound (3).

The extent to which CT/MRI are being utilized for HCC surveillance in the United States and their impact on overall survival (OS) in patients with HCC remain unknown. Therefore, we aimed to estimate the utilization of ultrasound, CT/MRI within 3 years prior to HCC diagnosis and investigate its association with OS in a population-based cohort.

## Materials and Methods

### Study Population

We conducted a population-based cohort study using the Surveillance Epidemiology and End Results (SEER)-Medicare database. The linked SEER-Medicare database combines demographic, clinical, and survival information for patients with cancer from the SEER program of cancer registries with Medicare claims information on covered health services from the time of Medicare eligibility until death (17, 18). The SEER program collects data on incident cancer cases from 18 cancer registries, including state, central, metropolitan, and the Alaska Native registries, which cover 28% of the United States (17, 19, 20). Medicare is the primary health insurer for approximately 94% of individuals age 65 years and older, and roughly 90% of Medicare beneficiaries are covered by both Part A (inpatient hospitalizations, skilled nursing facility stays, home health visits, and hospice care) and Part B (outpatient visits and physician office visits/services) benefits (21).

We included all Medicare beneficiaries, ages 68 years and older, who have been diagnosed with HCC [International Classification of Diseases (ICD)-Oncology-3 codes, site: C22.0 AND histology: 8170-8175] from 2011 to 2015. We limited the cohort to individuals ages 68 years and older to ensure 3 years of ascertainment for identification of risk factors and surveillance receipt. We excluded: (i) HCC cases ascertained by direct visualization without microscopic confirmation, or death certificate only; (ii) patients with Medicare Part A and B continuous enrollment fewer than 3 years; (iii) patients with less than 6-month follow-up time after HCC diagnosis to ensure complete capture of HCC-directed treatment; and (iv) patients enrolled in Medicare health maintenance organization plans as these plans were not required to submit individual claims information for services to the Centers for Medicare and Medicaid Services.

This study was conducted in accordance with Declaration of Helsinki and was approved by the NCI and a representative of the SEER registries for the use of the SEER-Medicare data.

### Study Variables

Variables of interest included sex, age, race/ethnicity [defined as non-Hispanic White, Black, Asian/Pacific Islander (API)/Others, and Hispanic], etiology of HCC, extent of tumor, types of HCC treatment, NCI comorbidity index, the presence of diabetes, cirrhosis, ascites, and hepatic encephalopathy, surveillance for HCC, SEER region stratified by census tract poverty level, metropolitan/nonmetropolitan counties.

We extracted data on abdominal ultrasound and contrast-enhanced cross-sectional images (CT, MRI) within 3 years prior to HCC diagnosis, excluding imaging for the same month of HCC diagnosis (22, 23). Receipt of abdominal ultrasound (76700 or 76705), contrast-enhanced CT (74160, 74170, or 74177), or MRI (74182 or 74183) was identified using the Current Procedural Terminology codes. Patients who had at least one CT/MRI within 3 years before HCC diagnosis were classified as the CT/MRI group. Patients who had at least one ultrasound, without CT/MRI, within 3 years before HCC diagnosis were classified as the ultrasound group. Patients without any ultrasound, CT, or MRI within 3 years prior to HCC diagnosis were classified as no imaging group. Proportion of time covered (PTC) was defined as the proportion of the 36-month study period in which patients had received abdominal imaging, with each imaging study providing 7 months of coverage (month of imaging + following 6 months; refs. 22, 24). For patients receiving imaging within 7 months before HCC diagnosis, the number of months of coverage were calculated from imaging to HCC diagnosis.

To define liver disease etiology, Medicare claims ICD, 9th revision or 10th revision codes were used for hepatitis C virus (HCV), hepatitis B virus (HBV), alcoholic liver disease (ALD), NAFLD, and others. As NAFLD is often under-coded, patients with ICD-9 or 10 code for obesity, diabetes, history of bariatric surgery or both dyslipidemia and hypertension in the absence of HBV, HCV, alcohol abuse, and other known liver disease were also classified as NAFLD (23). For patients with multiple etiologies, etiology was classified with the following hierarchy (HCV > HBV > ALD > others > NAFLD).

Cirrhosis was defined on the basis of ICD-9 or ICD-10 codes from Medicare claims (25). We used diagnosis and procedure codes 1 year before HCC diagnosis to calculate the NCI comorbidity index as a measure of noncancer comorbidity (26). The NCI comorbidity index was calculated after excluding liver conditions and diabetes to avoid collinearity in multivariable models (23).

Tumor characteristics were extracted from the SEER Patient Entitlement and Diagnosis Summary File. As SEER only provides the number of tumor nodules as a binary variable (unifocal vs. multifocal), we defined early-stage HCC as a single tumor, less than or equal to 5 cm in diameter without vascular invasion or extrahepatic metastasis, as we have previously done (23, 27).

### Statistical Analysis

Baseline demographic and clinical characteristics were summarized by standard descriptive measures (frequency and percentage for categorical variables and mean ± SD for continuous variables). These characteristics were then compared using Pearson *χ*^2^ test for categorical variables and the *t* test or one-way ANOVA for continuous variables as appropriate.

Patients who had no follow-up period after diagnosis or died during the same calendar month of HCC diagnosis (0 month follow-up) were excluded from the survival analysis (*n* = 710). Sensitivity analysis was performed after including those 710 patients who were excluded in the survival analysis. Survival probabilities were estimated using the Kaplan–Meier method and compared using the log-rank test. Cox regression was used to investigate the impact of imaging on OS after adjusting for demographic and clinical characteristics. Two separate multivariable models were developed: one with receipt of any imaging, stratified by modality group (CT/MRI, ultrasound, no imaging) and the other with PTC by ultrasound and CT/MRI. Fine-Gray subdistribution hazard model was used to analyze both HCC-specific and non-HCC deaths, regressing the hazard of death while adjusting for demographic and clinical characteristics (28).

Lead- and length-time biases were corrected using the method proposed by Duffy and colleagues (29, 30) Lead time is the time between early cancer detection by screening and when cancer otherwise would present symptomatically, which can lead to perceived survival benefit even if the disease course was not changed. Statistical correction for lead-time bias is based on sojourn time (1/λ), the period during which HCC is asymptomatic but screen-detectable. Following the parametric model by Duffy and colleagues, correction for lead-time bias involves estimation of the additional follow-up time due to lead time in the case of screen-detected cancer (29). The expected additional follow-up time, *s*, is 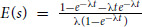 for a patient with screen-detected cancer known to be dead at time *t* after diagnosis and 
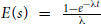
 for a patient with screen-detected cancer known to be alive at time *t* after diagnosis. To correct for lead-time bias, *E*(*s*) was subtracted from observed survival time of screen-detected patients, which were defined as those who received screening within 6 months prior to HCC diagnosis (29). We assumed an exponential distribution for the sojourn time 1/λ with a mean of 6 months for our base-case analysis, based on prior studies (31–33); however, we also performed sensitivity analyses with the mean sojourn times of 3 and 9 months.

Length-biased time relates to slow-growing tumors, which are less likely to be fatal, also have a longer asymptomatic period, and therefore are more likely to be screen-detected. Length-time bias was adjusted on the basis of the proportion of patients with slow-growing tumors and the relative risk of death from slow-growing tumors versus aggressive tumors following the method proposed by Duffy and colleagues (29). The relative risk of death from screen-detected versus symptomatic tumors, φ, was estimated by the observed probability of death from screen-detected, 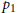, and symptomatic tumors, 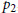, and the observed probability of screen-detected tumors, 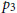, by giving plausible values for a proportion of patients with slow-growing tumors, 1 – *q*, and the relative risk of death from slow-growing tumors versus aggressive tumors, θ, that is: 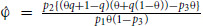.

We assumed that 20% of HCCs are slow-growing for our base-case analysis (34, 35), and we performed sensitivity analyses with proportions of 10% and 30%. For this range of values, plausible values for the relative risk of death from slow-growing tumors versus aggressive tumors were 0.8 and 0.9. We used 0.9 as our base case, and 0.8 as a sensitivity analysis. Thus, in total, we tested six scenarios for length-time bias adjustment.

All statistical analyses were performed using SAS 9.4 (SAS Institute, Inc.) and Stata 16.1 (StataCorp) software with two-sided tests and a significance level of 0.05.

### Data Availability

We used the SEER-Medicare Linked Database to generate the data in this study. The SEER-Medicare Linked Database is not publicly available due to the sensitive nature of the data and risk of reidentification. Investigators are required to obtain approval from SEER-Medicare to obtain the data.

## Results

### Patient Characteristics

Clinical characteristics of the 5,098 eligible patients with HCC are described in Table 1. About two-thirds of patients were male and the mean age was 76.8 years. The cohort was racially diverse with two-thirds non-Hispanic White, 16% API/Others, 13% Hispanic, and 8% non-Hispanic Black. Most patients had low NCI comorbidity scores, with only 10% having high comorbidity. About three-fourths had underlying cirrhosis, and the most common etiologies of liver disease were NAFLD, hepatitis C, and alcohol-associated liver disease. Only 19% of patients had early-stage HCC, and only 23% of patients underwent curative-intent therapies.

**TABLE 1:** Demographic and clinical characteristics of patients with HCC

Characteristics	Total (*n* = 5,098)	US (*n* = 1,077)	CT/MRI (*n* = 2,216)	No imaging (*n* = 1,805)	*P*
**Male sex, *n* (%)**	3,438 (67.4)	694 (64.4)	1,440 (65.0)	1,304 (72.2)	<0.001
**Age, mean (SD)**	76.8 (6.2)	76.8 (6.3)	75.9 (5.9)	77.7 (6.4)	<0.001
**Race/ethnicity, *n* (%)**	—	—	—	—	<0.001
Non-Hispanic White	3,224 (63.2)	645 (59.9)	1,371 (61.9)	1,208 (66.9)	—
Non-Hispanic Black	390 (7.7)	71 (6.6)	157 (7.1)	162 (9.0)	—
Non-Hispanic API/Others	810 (15.9)	202 (18.8)	364 (16.4)	244 (13.5)	—
Hispanic	674 (13.2)	159 (14.8)	324 (14.6)	191 (10.6)	—
**Census poverty level, *n* (%)**	—	—	—	—	0.32
0% to <5% poverty	1,011 (19.8)	211 (19.6)	465 (21.0)	335 (18.6)	—
5% to <10% poverty	1,192 (23.4)	236 (21.9)	528 (23.8)	428 (23.7)	—
10% to <20% poverty	1,560 (30.6)	341 (31.7)	647 (29.2)	572 (31.7)	—
20% to 100% poverty	1,335 (26.2)	289 (26.8)	576 (26.0)	470 (26.0)	—
**Rural-Urban counties, *n* (%)**	—	—	—	—	0.02
Metro > 1 million	3,029 (59.4)	657 (61.0)	1,348 (60.8)	1,024 (56.7)	—
Metro 250k to 1 million	1,028 (20.2)	218 (20.3)	437 (19.7)	373 (20.7)	—
Metro < 250k	414 (8.1)	80 (7.4)	187 (8.5)	147 (8.1)	—
Non-Metro/Rural	627 (12.3)	122 (11.3)	244 (11.0)	261 (14.5)	—
**NCI comorbidity index, *n* (%)**	—	—	—	—	<0.001
Low (0 to 2)	3,842 (75.4)	759 (70.5)	1,662 (75.0)	1,421 (78.7)	—
Moderate (>2 to 4)	725 (14.2)	177 (16.4)	323 (14.6)	225 (12.5)	—
High (>4)	531 (10.4)	141 (13.1)	231 (10.4)	159 (8.8)	—
**Etiology**	—	—	—	—	<0.001
HCV	1,715 (33.6)	404 (37.5)	915 (41.3)	396 (21.9)	—
NAFLD	1,813 (35.6)	339 (31.5)	628 (28.3)	846 (46.9)	—
ALD	895 (17.6)	206 (19.1)	416 (18.8)	273 (15.1)	—
HBV	246 (4.8)	68 (6.3)	104 (4.7)	74 (4.1)	—
Others/None	429 (8.4)	60 (5.6)	153 (6.9)	216 (12.0)	—
**Diabetes, *n* (%)**	3,262 (63.9)	738 (68.5)	1,471 (66.4)	1,053(58.3)	<0.001
**Cirrhosis, *n* (%)**	3,815 (74.8)	883 (82.0)	1,802 (81.3)	1,130 (62.6)	<0.001
**Ascites, *n* (%)**	2,213 (43.4)	500 (46.4)	1,002 (45.2)	711 (39.4)	<0.001
**Hepatic encephalopathy, *n* (%)**	875 (17.2)	201 (18.7)	474 (21.4)	200 (11.1)	<0.001
**Early-stage HCC** ^a^ **, *n* (%)**	967 (19.0)	214 (19.9)	572 (25.8)	181 (10.0)	<0.001
**Treatment type, *n* (%)**	—	—	—	—	<0.001
Curative treatment	—	—	—	—	—
Tumor ablation	589 (11.6)	136 (12.6)	376 (17.0)	77 (4.3)	—
Liver resection	467 (9.2)	96 (8.9)	236 (10.7)	135 (7.5)	—
Liver transplant	111 (2.2)	19 (1.8)	77 (3.5)	15 (0.8)	—
Noncurative treatment	—	—	—	—	—
Chemoembolization	777 (15.2)	169 (15.7)	399 (18.0)	209 (11.6)	—
Radioembolization	225 (4.4)	54 (5.0)	107 (4.8)	64 (3.6)	—
Other radiation	132 (2.6)	33 (3.1)	61 (2.7)	38 (2.1)	—
Systemic treatment	675 (13.2)	130 (12.1)	306 (13.8)	239 (13.2)	—
Others/best supportive care	2,122 (41.6)	440 (40.8)	654 (29.5)	1,028 (56.9)	—

Abbreviations: ALD, alcoholic liver disease; API, Asian/Pacific Islander; CT, computed tomography; HBV, hepatitis B virus; HCC, hepatocellular carcinoma; HCV, hepatitis C virus; Metro, metropolitan; MRI, magnetic resonance imaging; NAFLD, nonalcoholic fatty liver disease; NCI, National Cancer Institute; SD, standard deviation; TACE, transarterial chemoembolization; TARE, transarterial radioembolization; US, ultrasound.

^a^Single lesion ≤5 cm without vascular invasion or metastasis.

### Receipt and Correlates of Abdominal Imaging Prior to HCC Diagnosis

Abdominal imaging was observed in 3,293 (65%) patients within 3 years prior to HCC diagnosis. Of those with imaging, 2,216 (67%) were in CT/MRI group and 1,077 (33%) were in the ultrasound group. Mean time interval between first imaging (ultrasound or CT/MRI) and HCC diagnosis was 560.5 days (SD: 396.7 days). Among 2,216 patients in CT/MRI group, 658 patients had only CT/MRI during the 3 years prior to HCC diagnosis without any ultrasound. There were 440 patients who had a CT/MRI first and an ultrasound later and 1,040 patients who had ultrasound first and CT/MRI later and 78 patients who had first ultrasound and CT/MRI at the same time. For patients who had ultrasound before CT/MRI, median time between the performance of ultrasound and the CT/MRI was 194.5 days [interquartile range (IQR): 22–584 days]. For patients who had CT/MRI before ultrasound, median time between the performance of ultrasound and the CT/MRI was 230.5 days (IQR: 61–553.5 days).

Younger age, female sex, hepatitis C etiology, diabetes, presence of cirrhosis, and presence of hepatic encephalopathy was associated with receipt of CT/MRI (Supplementary Table S1). Higher proportion of patients in the CT/MRI group presented with early-stage HCC and received potentially curative treatment than patients in the ultrasound or no imaging group (Table 1).

**FIGURE 1:**
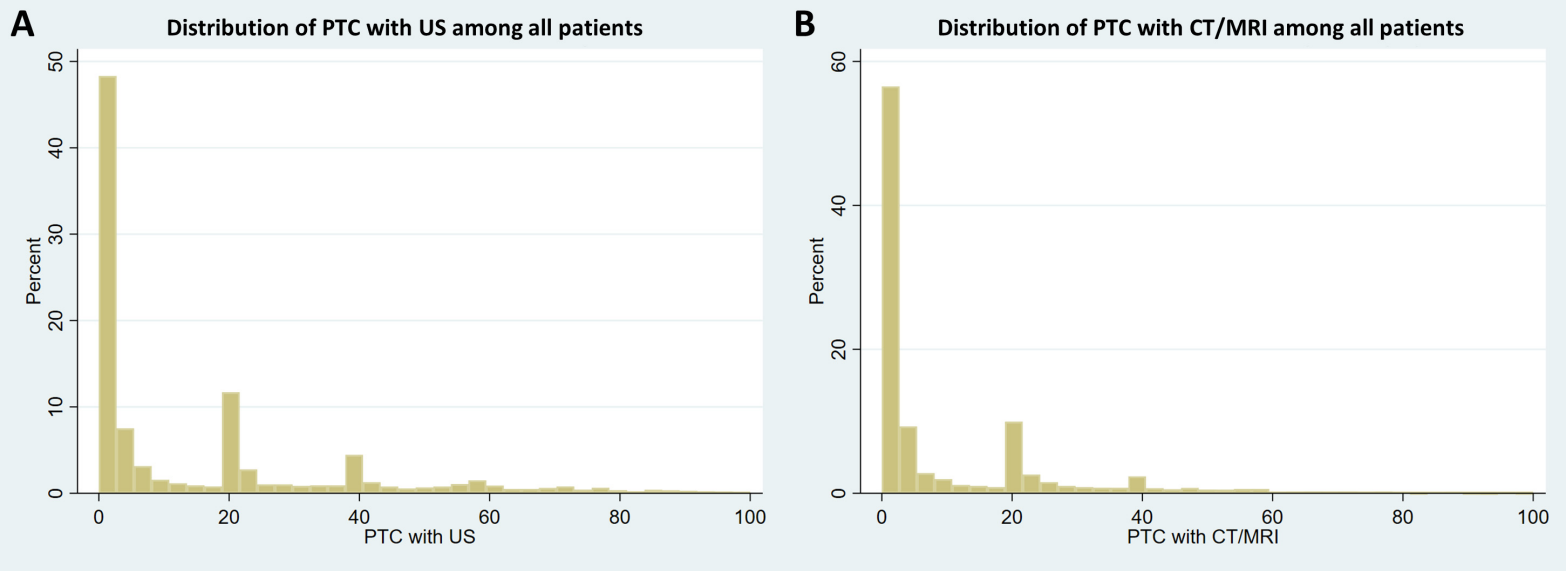
Distribution of PTC with ultrasound (US) or CT/MRI for patients with HCC. **A,** Distribution of PTC with ultrasound for all the patients with HCC in the study. **B,** Distribution of PTC with CT/MRI for all the patients with HCC in the study. CT, computed tomography; HBV, hepatitis B virus; HCC, hepatocellular carcinoma; MRI, magnetic resonance imaging; PTC, proportion of time covered.

Median PTC of the 36-month period prior to HCC diagnosis by any abdominal imaging (ultrasound, CT, or MRI) was only 5.6% (IQR: 0%–36%). Only one in ten patients had PTC > 50% using ultrasound (9.8%; Fig. 1A) and 1 in 20 patients had PTC > 50% using CT/MRI (4.2%; Fig. 1B).

### Association of Abdominal Imaging with OS

**FIGURE 2:**
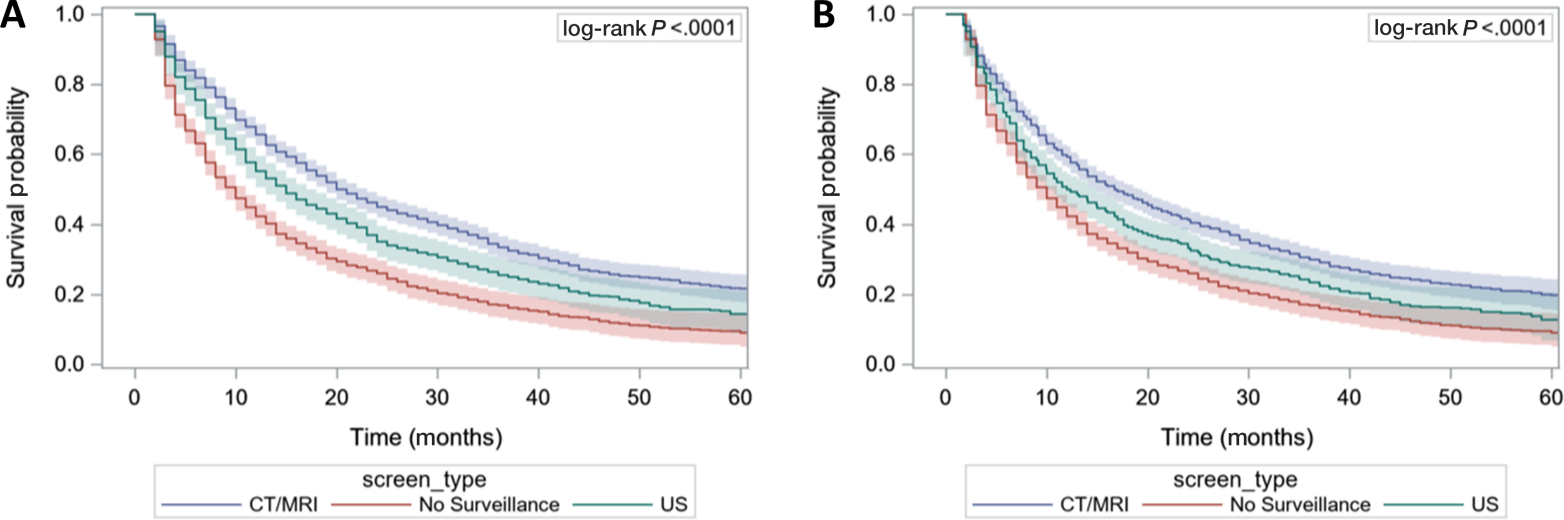
OS estimates by Imaging group. Comparison of OS between patients with HCC receiving different types of imaging without adjustment for lead-time bias (**A**) and after adjustment for lead-time bias with mean sojourn time of 6 months (**B**). Median OS was 20.5 months (95% CI: 19–22 months), 15 months (95% CI: 13–17 months), and 10 months (95% CI: 9–11 months) for CT/MRI, US, and no imaging, respectively without adjustment for lead-time bias. Median OS was 16.7 months (95% CI: 15.8–18.6 months) and 12 months (95% CI: 10.7–14.1 months) for CT/MRI and US, respectively, with adjustment for lead-time bias. Lead-time adjustment would not affect median OS of no imaging group. CT, computed tomography; HCC, hepatocellular carcinoma; MRI, magnetic resonance imaging; US, ultrasound.

The median OS of the entire cohort was 15 months [95% confidence interval (CI): 14–16 months]. One- and 3-year survival probabilities were 66% (95% CI: 63–68) and 33% (95% CI: 31–36) for the CT/MRI group; 55% (95% CI: 52–58) and 26% (95% CI: 23–28) for ultrasound group; and 42% (95% CI: 40–45) and 17% (95% CI: 15–19) for the no imaging group (Fig. 2A), respectively. In multivariable Cox regression analysis (Table 2), compared with no imaging group, the ultrasound [adjusted HR (aHR): 0.87, 95% CI: 0.79–0.95] and CT/MRI (aHR: 0.68, 95% CI: 0.63–0.74) groups were both associated with improved survival. Compared with the ultrasound group, CT/MRI group was associated with improved survival (aHR: 0.79, 95% CI: 0.72–0.86). PTC with ultrasound and CT/MRI were also both associated with improved survival, with a larger effect size observed with CT/MRI (aHR per 10%: 0.93, 95% CI: 0.91–0.95) than ultrasound (aHR per 10%: 0.96, 95% CI: 0.95–0.98).

**TABLE 2:** Factor associated with OS for patients with HCC

	Univariate analysis		Multivariable analysis-1^b^		Multivariable analysis-2^b^	
Characteristics	HR (95% CI)	*P*	aHR (95% CI)	*P*	aHR (95% CI)	*P*
**Female sex (Ref. Male)**	0.86 (0.80–0.92)	<0.001	0.92 (0.85–0.99)	0.03	0.93 (0.87–1.01)	0.07
**Age**	1.03 (1.02–1.03)	<0.001	1.02 (1.01–1.02)	<0.001	1.02 (1.01–1.02)	<0.001
**Race/ethnicity**	—	—	—	—	—	—
Non-Hispanic White	Ref	Ref	Ref	Ref	Ref	Ref
Non-Hispanic Black	1.01 (0.89–1.15)	0.86	0.99 (0.86–1.14)	0.89	0.99 (0.86–1.13)	0.87
Non-Hispanic API/Others	0.65 (0.59–0.72)	<0.001	0.82 (0.74–0.91)	<0.001	0.83 (0.75–0.93)	0.001
Hispanic	0.94 (0.85–1.04)	0.22	0.88 (0.79–0.97)	0.01	0.87 (0.79–0.97)	0.009
**Census poverty level**	—	—	—	—	—	—
<5%	Ref	Ref	Ref	Ref	Ref	Ref
5% to <10%	1.14 (1.03–1.26)	0.01	1.06 (0.96–1.18)	0.25	1.05 (0.95–1.17)	0.31
10% to <20%	1.12 (1.02–1.24)	0.02	1.05 (0.95–1.15)	0.37	1.05 (0.95–1.16)	0.35
20% to 100%	1.14 (1.03–1.26)	0.009	1.11 (1.00–1.24)	0.052	1.11 (1.00–1.24)	0.051
**Rural-Urban**	—	—	—	—	—	—
Metro > 1 million	Ref	Ref	Ref	Ref	Ref	Ref
Metro 250k to 1 million	1.15 (1.06–1.25)	0.001	1.15 (1.06–1.26)	0.001	1.15 (1.06–1.26)	0.001
Metro < 250k	1.23 (1.09–1.39)	0.001	1.05 (0.92–1.19)	0.49	1.03 (0.91–1.17)	0.63
Non-Metro/Rural	1.28 (1.15–1.42)	<0.001	1.10 (0.99–1.22)	0.09	1.08 (0.97–1.21)	0.15
**NCI comorbidity index**	—	—	—	—	—	—
Low (0 to 2)	Ref	Ref	Ref	Ref	Ref	Ref
Moderate (>2 to 4)	1.23 (1.11–1.35)	<0.001	1.08 (0.98–1.19)	0.12	1.07 (0.97–1.17)	0.19
High (>4)	1.60 (1.43–1.78)	<0.001	1.28 (1.14–1.43)	<0.001	1.27 (1.14–1.42)	<0.001
**Etiology**	—	—	—	—	—	—
HCV	Ref	Ref	Ref	Ref	Ref	Ref
NAFLD	1.37 (1.27–1.49)	<0.001	1.21 (1.10–1.33)	<0.001	1.19 (1.09–1.31)	<0.001
ALD	1.36 (1.24–1.50)	<0.001	1.10 (1.00–1.22)	0.06	1.08 (0.98–1.20)	0.12
HBV	0.87 (0.73–1.03)	0.10	1.06 (0.89–1.27)	0.48	1.05 (0.88–1.25)	0.56
Others/None	1.21 (1.06–1.38)	0.004	1.10 (0.96–1.26)	0.19	1.10 (0.96–1.26)	0.19
**Diabetes**	1.09 (1.02–1.17)	0.01	1.00 (0.93–1.08)	0.93	1.02 (0.94–1.10)	0.66
**Cirrhosis**	1.06 (0.98–1.14)	0.16	0.99 (0.90–1.10)	0.86	0.99 (0.90–1.10)	0.91
**Ascites**	1.78 (1.67–1.91)	<0.001	1.83 (1.68–1.98)	<0.001	1.86 (1.71–2.02)	<0.001
**Hepatic encephalopathy**	1.54 (1.41–1.68)	<0.001	1.44 (1.31–1.58)	<0.001	1.49 (1.35–1.64)	<0.001
**Imaging type**	—	—	—	—	—	—
No imaging	Ref	Ref	Ref	Ref	—	—
US	0.75 (0.68–0.82)	<0.001	0.87 (0.79–0.95)	0.003	—	—
CT/MRI	0.58 (0.54–0.63)	<0.001	0.68 (0.63–0.74)	<0.001	—	—
**PTC**	—	—	—	—	—	—
US (per 10%)	0.92 (0.91–0.94)	<0.001	—	—	0.96 (0.95–0.98)	<0.001
CT (per 10%)	0.90 (0.88–0.92)	<0.001	—	—	0.93 (0.91–0.95)	<0.001
**Early-stage HCC** ^a^	0.46 (0.42–0.50)	<0.001	0.56 (0.51–0.62)	<0.001	0.57 (0.52–0.63)	<0.001
**Curative treatment**	0.27 (0.25–0.30)	<0.001	0.32 (0.29–0.35)	<0.001	0.31 (0.29–0.34)	<0.001

Abbreviations: aHR, adjusted hazard ratio; ALD, alcoholic liver disease; API, Asian/Pacific Islander; CT, computed tomography; HBV, hepatitis B virus; HCC, hepatocellular carcinoma; HCV, hepatitis C virus; HR, hazard ratio; Metro, metropolitan; MRI, magnetic resonance imaging; NAFLD, nonalcoholic fatty liver disease; NCI, National Cancer Institute; US, ultrasound.

^a^Single lesion ≤5 cm without vascular invasion or metastasis.

^b^Model 1 included imaging category while model 2 include PTC in multivariable Cox regression.

Survival analysis showed consistent results after including 710 patients without follow-up or death within 1 month after HCC diagnosis. In multivariable Cox regression analysis, compared with no imaging, the ultrasound (aHR: 0.85, 95% CI: 0.78–0.93) and CT/MRI (aHR: 0.65, 95% CI: 0.60–0.70) groups were both associated with improved survival. PTC with ultrasound and CT/MRI were also both associated with improved survival, with a larger effect size observed with CT/MRI (aHR per 10%: 0.92, 95% CI: 0.90–0.94) than ultrasound (aHR per 10%: 0.96, 95% CI: 0.94–0.97). When the imaging group was redefined only taking into consideration of the tests within 1–7 months before HCC diagnosis, association between imaging modality and OS remains consistent: compared with no imaging, the ultrasound (aHR: 0.89, 95% CI: 0.81–0.97) and CT/MRI (aHR: 0.73, 95% CI: 0.68–0.79) groups were both associated with improved survival.

We next investigated the association between abdominal imaging and HCC-specific and non–HCC-specific mortality. After excluding 340 patients without information on the cause of death, the analysis included a total of 4,048 patients. Of these patients, 22% (*n* = 876) patients were alive at the end of the study period, while 54% (*n* = 2,181) had died because of HCC. In addition, 4% (*n* = 163) died from other liver diseases and 20% (*n* = 828) died from other causes. The multivariable subdistribution hazard model revealed that, compared with no imaging group, ultrasound (aHR: 0.83, 95% CI: 0.73–0.94) and CT/MRI (aHR: 0.77, 95% CI: 0.70–0.86) groups had a decreased relative incidence of HCC-specific death. However, we found that both the ultrasound (aHR: 1.18, 95% CI: 0.99–1.40) and CT/MRI (aHR: 0.89, 95% CI: 0.77–1.02) groups had no significant effect on the subdistribution hazard of non-HCC death, when compared with no imaging group. PTC with ultrasound (aHR per 10%: 0.96, 95% CI: 0.94–0.98) and CT/MRI (aHR per 10%: 0.94, 95% CI: 0.91–0.97) also both had a decreased relative incidence of HCC-specific death. However, we found that both the ultrasound (aHR per 10%: 1.01, 95% CI: 0.98–1.04) and CT/MRI (aHR per 10%: 1.02, 95% CI: 0.98–1.05) groups had no significant effect on the subdistribution hazard of non-HCC death, when compared with no imaging group, confirming that abdominal imaging prior to HCC diagnosis is associated with improved survival via reducing HCC-specific mortality.

The median OS and survival probabilities of patients with HCC after adjustment for lead- and length-time biases are summarized in Fig. 2B and Table 3. One-, 3-, and 5-year survival probabilities of patients in the CT/MRI group were significantly higher than patients in the ultrasound or no imaging groups, regardless of the assumption of sojourn times. After adjusting for lead-time bias, multivariable Cox regression analysis (Supplementary Table S2) showed that patients in the CT/MRI group experienced improved survival compared with no imaging group (aHR: 0.80, 95% CI: 0.74–0.87); however, the survival benefit in the ultrasound group was no longer observed (aHR: 1.00, 95% CI: 0.91–1.10). CT/MRI group experienced improved survival compared with ultrasound group after adjusting for the same covariates in Supplementary Table S2 and lead-time bias (aHR: 0.80, 95% CI: 0.74–0.88).

**TABLE 3:** Survival probabilities of patients with HCC by imaging group after adjustment for lead- and length-time bias

Imaging type	1-year survival (%) (95% CI)^a^	3-year survival (%) (95% CI)^a^	5-year survival (%) (95% CI)^a^	log-rank test
No imaging^b^	42 (40–45)^c^	17 (15–19)^c^	9 (7–11)^c^	—
**Unadjusted for lead-time bias**	—	—	—	<0.001
US	55 (52–58)^c^	26 (23–28)^c^	14 (11–17)^c^	—
CT/MRI	66 (63–68)^c^	33 (31–36)^c^	22 (20–24)^c^	—
**Adjusted for lead-time bias**	—	—	—	—
Mean sojourn time = 3 months	—	—	—	<0.001
US	53 (49–56)^c^	25 (22–27)^c^	13 (11–16)	—
CT/MRI	62 (60–64)^c^	32 (30–34)^c^	21 (18–23)^c^	—
Mean sojourn time = 6 months	—	—	—	<0.001
US	50 (47–53)^c^	24 (21–27)^c^	12 (10–15)	—
CT/MRI	59 (57–61)^c^	30 (28–33)^c^	20 (18–22)^c^	—
Mean sojourn time = 9 months	—	—	—	<0.001
US	49 (46–52)^c^	22 (20–25)^c^	12 (9–15)	—
CT/MRI	58 (55–60)^c^	29 (26–31)^c^	19 (17–21)^c^	—
**Adjusted for lead- and length-time bias**	—	—	—	—
Mean sojourn time = 3 months	—	—	—	—
US	51 (48–53)^c^	24 (21–26)^c^	13 (10–15)	—
CT/MRI	60 (58–62)^c^	31 (28–33)^c^	20 (17–21)^c^	—
Mean sojourn time = 6 months				—
US	49 (46–51)^c^	23 (20–25)^c^	12 (9–14)	—
CT/MRI	57 (55–58)^c^	30 (26–31)^c^	19 (15–20)^c^	—
Mean sojourn time = 9 months				—
US	48 (46–49)^c^	22 (19–23)^c^	12 (9–13)	—
CT/MRI	56 (54–57)^c^	28 (24–29)^c^	18 (15–20)^c^	—

Abbreviations: CI, confidence interval; CT, computed tomography; HCC, hepatocellular carcinoma; MRI, magnetic resonance imaging; US, ultrasound.

^a^For lead- and length-time bias adjustment, ranges of values from sensitivity analyses are given instead of 95% CIs.

^b^Survival estimates for no surveillance group were not changed as adjusting lead- or length-time bias.

^c^95% confidence intervals are not overlapped between each surveillance type.

## Discussion

In this large population-based cohort study of U.S. Medicare beneficiaries, we found abdominal imaging was underutilized within 3 years prior to HCC diagnosis. Only 1 in 10 patients had PTC ≥ 50% using ultrasound and 1 in 20 patients had PTC ≥ 50% using CT/MRI. Notably, CT/MRI imaging was associated with better OS in fully adjusted models, although the survival benefit of ultrasound-based imaging was mitigated after adjusting for lead- and length-time biases.

Overall prognosis of HCC is worse than other solid cancer in part due to lower surveillance implementation and presentation at an advanced stage disease. HCC surveillance is underutilized in the United States and it is recognized as one of the most critical performance gaps across the HCC care continuum (36). A recent meta-analysis of 29 studies, with a total of 118,799 patients reported a pooled estimate for surveillance use of 24.0% (19). Surveillance estimate in the population-based studies was even lower that only 8.8% of HCCs were detected under surveillance (19). Our PTC analysis showed that ultrasound is still the dominant form of imaging and it is being used more than twice compared with CT/MRI within 3 years prior to HCC diagnosis. Recently, Singal and colleagues summarized a conceptual model for the HCC screening continuum and the reasons for surveillance failure (37). One of the key reasons for the low surveillance in HCC compared with other cancers (e.g., breast or colon cancer) is difficulty with the identification of high-risk populations (38, 39). Multiple studies showed that lower surveillance receipt among at-risk individuals is in part due to higher prevalence of unrecognized cirrhosis (26, 40). Better recognition of cirrhosis via laboratory and imaging-based algorithms may improve the identification of candidates for surveillance implementation.

Accuracy of ultrasound for early-stage HCC detection is limited. A recent meta-analysis showed that ultrasound has 53% sensitivity for detection of early-stage HCC (6). Serum alpha-fetoprotein (AFP) can augment the accuracy of surveillance test but a combination of AFP and ultrasound still has inadequate performance with a 63% sensitivity for early-stage HCC detection (6). This is far lower than the sensitivity of mammogram (70%–90%) for breast cancer (41) and colonoscopy (>95%) for colon cancer detection (42), highlighting a need to have better surveillance tests. This is particularly important as obesity, metabolic syndrome and alcohol-associated liver diseases, predictors of inadequate ultrasound, have been in rapid increase (43), thus the performance of ultrasound for HCC surveillance will likely further decrease. Surveillance with cross-sectional images, particularly with MRI has been getting more attention recently (10). A single-center prospective surveillance study from Korea of 407 patients with cirrhosis compared the accuracy of ultrasound and liver-specific contrast-enhanced MRI for early-stage HCC detection. Compared with ultrasound, MRI had a higher HCC detection rate (86% vs. 28%, *P* < 0.001) and lower false-positive findings (3.0% vs. 5.6%; *P* = 0.004; ref. 11). Subsequent cost-effectiveness studies also showed that MRI-based surveillance is cost-effective (15, 16). Currently, high cost and long scanning time would be a significant barrier for wider adoption of MRI-based surveillance (44). Recently, abbreviated MRI was studied as an attractive option for HCC surveillance by shortening the scanning time with fewer magnetic resonance sequences. Compared with ultrasound, abbreviated MRI was shown to have higher sensitivity (86% vs. 28%, *P* < 0.001) at specificity >95% for detection of HCC (45). With a lower cost and high accuracy of MRI-based surveillance, indication and implementation for MRI-based surveillance will likely increase in the near future. It should also be noted that cost-effectiveness of such screening will be highly contingent on the medical system. Implementing a low cost streamlined CT/MRI screening could be more effective in the ultrasound with multipayer systems when it is being mandated by law, similar to mammography for breast cancer, to improve the cost-effectiveness of CT/MRI surveillance.

Surveillance with cross-sectional images will likely improve early HCC detection and a higher likelihood of curative treatment, thus improving OS. However, the potential limitation of CT/MRI surveillance should be carefully considered. For example, it can lead to overdiagnosis of HCC, defined as detecting clinically insignificant, indolent disease that would not impact lifespan expectancy (46). This potential is particularly true for detection and diagnosis of tumors between 1 and 2 cm, for which the positive predictive value of imaging characteristics is lower than that of larger tumors. This could lead to overtreatment, resulting in increased costs, as well as physical side effects, psychologic harms, and poorer quality of life (46). It may increase physical and psychologic harm with indeterminate findings that might lead to repeated intravenous contrast images or percutaneous liver biopsy (47, 48). Compare with other cancers with well-established screening program, net benefit of HCC surveillance program has not been studied rigorously in HCC, but these should be further investigated as we anticipate increasing diagnosis of HCC with effective HCC surveillance programs.

We acknowledge that our study has several limitations. First, SEER-Medicare lacks granularity, including data on liver disease severity (e.g., model for end-stage liver disease, Child-Pugh score) which may affect the indication for HCC surveillance. However, we captured complications of cirrhosis (ascites, hepatic encephalopathy) using ICD-9 or ICD-10 codes. Second, we examined abdominal imaging within 3 years prior to HCC diagnosis as a surrogate for HCC surveillance test, according to our previous publication (22, 23), although this operational definition is not concordant with guideline recommendations for semiannual surveillance. We used this definition given the low proportion of patients with any imaging during the study period. To address this limitation, we also measured PTC to consider surveillance as a continuum with various degrees of compliance. Study population in this study is heterogeneous in terms of the way and method of HCC surveillance due to the retrospective nature of the study. However, this reflects the real-life practice of HCC surveillance program in the U.S. general population and the results provide preliminary evidence to conduct prospective randomized controlled trials to address this question. Third, intent of imaging could not be determined in this study given the retrospective nature of the study and many imaging studies were likely performed for nonsurveillance purposes. In the same vein, Current Procedural Terminology codes could not capture more granular clinical data including a specific type of intravenous contrast agent and contrast sequences. Although we have only counted CT/MRI with intravenous contrast, these abdominal images (e.g., CT/MRI for evaluation of abdominal pain using single portal venous phase contrast CT) would not have been optimized for early HCC detection, thus potential survival benefit of CT/MRI surveillance could have been underestimated in the current study.

Finally, Medicare population represents older individuals and the study results might not be generalizable to younger patients with HCC. Despite these limitations, this is the largest contemporary cohort of patients that describes the utilization of CT/MRI prior to the diagnosis of HCC and its association with OS.

In conclusion, abdominal imaging is severely underutilized prior to HCC diagnosis among Medicare beneficiaries in the United States. While both ultrasound and CT/MRI group were associated with better survival compared with no imaging, the results suggest that CT/MRI surveillance may have potential survival benefit compared with ultrasound surveillance. Given the increasing burden of a high-risk population with obesity and metabolic liver diseases, the accuracy of ultrasound as a surveillance test will likely decline. Therefore, the role of CT/MRI surveillance, its cost-effectiveness, and harms relative to ultrasound should be further investigated in a randomized controlled trial.

## Supplementary Material

Supplementary Table S1 Supplementary Table S1 shows the demographic and clinical factors associated with receipt of MRI/CT test in HCC patients.

Supplementary Table S2 Supplementary Table S2 shows the demographic and clinical factors associated with overall survival for HCC patients after adjusting for lead-time bias.

## References

[1] Yang JD , Hainaut P, Gores GJ, Amadou A, Plymoth A, Roberts LR. A global view of hepatocellular carcinoma: trends, risk, prevention and management. Nat Rev Gastroenterol Hepatol 2019;16:589–604. 31439937 10.1038/s41575-019-0186-y PMC6813818

[2] Singal AG , Zhang E, Narasimman M, Rich NE, Waljee AK, Hoshida Y, . HCC surveillance improves early detection, curative treatment receipt, and survival in patients with cirrhosis: a meta-analysis. J Hepatol 2022;77:128–39. 35139400 10.1016/j.jhep.2022.01.023 PMC9232881

[3] Marrero JA , Kulik LM, Sirlin CB, Zhu AX, Finn RS, Abecassis MM, . Diagnosis, staging, and management of hepatocellular carcinoma: 2018 practice guidance by the american association for the study of liver diseases. Hepatology 2018;68:723–50. 29624699 10.1002/hep.29913

[4] European Association for the Study of the Liver. EASL clinical practice guidelines: management of hepatocellular carcinoma. J Hepatol 2018;69:182–236. 29628281 10.1016/j.jhep.2018.03.019

[5] Omata M , Cheng AL, Kokudo N, Kudo M, Lee JM, Jia J, . Asia-Pacific clinical practice guidelines on the management of hepatocellular carcinoma: a 2017 update. Hepatol Int 2017;11:317–70. 28620797 10.1007/s12072-017-9799-9 PMC5491694

[6] Tzartzeva K , Obi J, Rich NE, Parikh ND, Marrero JA, Yopp A, . Surveillance imaging and alpha fetoprotein for early detection of hepatocellular carcinoma in patients with cirrhosis: a meta-analysis. Gastroenterology 2018;154:1706–18. 29425931 10.1053/j.gastro.2018.01.064 PMC5927818

[7] Simmons O , Fetzer DT, Yokoo T, Marrero JA, Yopp A, Kono Y, . Predictors of adequate ultrasound quality for hepatocellular carcinoma surveillance in patients with cirrhosis. Aliment Pharmacol Ther 2017;45:169–77. 27862091 10.1111/apt.13841 PMC7207219

[8] Schoenberger H , Chong N, Fetzer DT, Rich NE, Yokoo T, Khatri G, . Dynamic changes in ultrasound quality for hepatocellular carcinoma screening in patients with cirrhosis. Clin Gastroenterol Hepatol 2022;20:1561–9. 34119640 10.1016/j.cgh.2021.06.012 PMC8660956

[9] Chong N , Schoenberger H, Yekkaluri S, Fetzer DT, Rich NE, Yokoo T, . Association between ultrasound quality and test performance for HCC surveillance in patients with cirrhosis: a retrospective cohort study. Aliment Pharmacol Ther 2022;55:683–90. 35170052 10.1111/apt.16779

[10] Lee YT , Fujiwara N, Yang JD, Hoshida Y. Risk stratification and early detection biomarkers for precision HCC screening. Hepatology 2022 [Online ahead of print]. 10.1002/hep.32779 PMC9995677 36082510

[11] Kim SY , An J, Lim YS, Han S, Lee JY, Byun JH, . MRI with liver-specific contrast for surveillance of patients with cirrhosis at high risk of hepatocellular carcinoma. JAMA Oncol 2017;3:456–63. 27657493 10.1001/jamaoncol.2016.3147 PMC5470420

[12] Yoon JH , Lee JM, Lee DH, Joo I, Jeon JH, Ahn SJ, . A comparison of biannual two-phase low-dose liver CT and US for HCC Surveillance in a group at high risk of HCC development. Liver Cancer 2020;9:503–17. 33083277 10.1159/000506834 PMC7548851

[13] Roberts LR , Sirlin CB, Zaiem F, Almasri J, Prokop LJ, Heimbach JK, . Imaging for the diagnosis of hepatocellular carcinoma: a systematic review and meta-analysis. Hepatology 2018;67:401–21. 28859233 10.1002/hep.29487

[14] Goossens N , Singal AG, King LY, Andersson KL, Fuchs BC, Besa C, . Cost-effectiveness of risk score-stratified hepatocellular carcinoma screening in patients with cirrhosis. Clin Transl Gastroenterol 2017;8:e101. 28640287 10.1038/ctg.2017.26 PMC5518949

[15] Kim HL , An J, Park JA, Park SH, Lim YS, Lee EK. Magnetic resonance imaging is cost-effective for hepatocellular carcinoma surveillance in high-risk patients with cirrhosis. Hepatology 2019;69:1599–613. 30365164 10.1002/hep.30330

[16] Nahon P , Najean M, Layese R, Zarca K, Segar LB, Cagnot C, . Early hepatocellular carcinoma detection using magnetic resonance imaging is cost-effective in high-risk patients with cirrhosis. JHEP Rep 2021;4:100390. 34977518 10.1016/j.jhepr.2021.100390 PMC8683591

[17] Warren JL , Klabunde CN, Schrag D, Bach PB, Riley GF. Overview of the SEER-Medicare data: content, research applications, and generalizability to the United States elderly population. Med Care 2002;40:IV-3-18. 10.1097/01.MLR.0000020942.47004.03 12187163

[18] Enewold L , Parsons H, Zhao L, Bott D, Rivera DR, Barrett MJ, . Updated overview of the SEER-medicare data: enhanced content and applications. J Natl Cancer Inst Monogr 2020;2020:3–13. 32412076 10.1093/jncimonographs/lgz029 PMC7225666

[19] Wolf E , Rich NE, Marrero JA, Parikh ND, Singal AG. Use of hepatocellular carcinoma surveillance in patients with cirrhosis: a systematic review and meta-analysis. Hepatology 2021;73:713–25. 32383272 10.1002/hep.31309 PMC7648722

[20] Hester CA , Rich NE, Singal AG, Yopp AC. Comparative analysis of nonalcoholic steatohepatitis- versus viral hepatitis- and alcohol-related liver disease-related hepatocellular carcinoma. J Natl Compr Canc Netw 2019;17:322–9. 30959469 10.6004/jnccn.2018.7105

[21] Centers for Medicare and Medicaid Services. CMS program statistics: 2017 Medicare enrollment section. Available from: https://data.cms.gov/collection/cms-program-statistics.

[22] Choi DT , Kum HC, Park S, Ohsfeldt RL, Shen Y, Parikh ND, . Hepatocellular carcinoma screening is associated with increased survival of patients with cirrhosis. Clin Gastroenterol Hepatol 2019;17:976–87. 30616961 10.1016/j.cgh.2018.10.031 PMC6431264

[23] Karim MA , Singal AG, Kum HC, Lee YT, Park S, Rich NE, . Clinical characteristics and outcomes of nonalcoholic fatty liver disease-associated hepatocellular carcinoma in the United States. Clin Gastroenterol Hepatol 2023;21:670–80. 35307595 10.1016/j.cgh.2022.03.010 PMC9481743

[24] Murphy CC , Halm EA, Skinner CS, Balasubramanian BA, Singal AG. Challenges and approaches to measuring repeat fecal immunochemical test for colorectal cancer screening. Cancer Epidemiol Biomarkers Prev 2020;29:1557–63. 32457184 10.1158/1055-9965.EPI-20-0230 PMC7416474

[25] Kramer JR , Davila JA, Miller ED, Richardson P, Giordano TP, El-Serag HB. The validity of viral hepatitis and chronic liver disease diagnoses in Veterans Affairs administrative databases. Aliment Pharmacol Ther 2008;27:274–82. 17996017 10.1111/j.1365-2036.2007.03572.x

[26] Yang JD , Ahmed Mohammed H, Harmsen WS, Enders F, Gores GJ, Roberts LR. Recent trends in the epidemiology of hepatocellular carcinoma in olmsted county, minnesota: a US population-based study. J Clin Gastroenterol 2017;51:742–8. 28445235 10.1097/MCG.0000000000000810 PMC5552490

[27] Wagle NS , Park S, Washburn D, Ohsfeldt RL, Rich NE, Singal AG, . Racial, ethnic, and socioeconomic disparities in curative treatment receipt and survival in hepatocellular carcinoma. Hepatol Commun 2022;6:1186–97. 34796703 10.1002/hep4.1863 PMC9035560

[28] Fine JP , Gray RJ. A proportional hazards model for the subdistribution of a competing risk. J Am Stat Assoc 1999;94:496–509.

[29] Duffy SW , Nagtegaal ID, Wallis M, Cafferty FH, Houssami N, Warwick J, . Correcting for lead time and length bias in estimating the effect of screen detection on cancer survival. Am J Epidemiol 2008;168:98–104. 18504245 10.1093/aje/kwn120

[30] Facciorusso A , Ferrusquía J, Muscatiello N. Lead time bias in estimating survival outcomes. Gut 2016;65:538–9. 26163490 10.1136/gutjnl-2015-310199

[31] El-Serag HB , Kramer JR, Chen GJ, Duan Z, Richardson PA, Davila JA. Effectiveness of AFP and ultrasound tests on hepatocellular carcinoma mortality in HCV-infected patients in the USA. Gut 2011;60:992–7. 21257990 10.1136/gut.2010.230508

[32] Thein HH , Campitelli MA, Yeung LT, Zaheen A, Yoshida EM, Earle CC. Improved survival in patients with viral hepatitis-induced hepatocellular carcinoma undergoing recommended abdominal ultrasound surveillance in Ontario: a population-based retrospective cohort study. PLoS One 2015;10:e0138907. 26398404 10.1371/journal.pone.0138907 PMC4580446

[33] Singal AG , Pillai A, Tiro J. Early detection, curative treatment, and survival rates for hepatocellular carcinoma surveillance in patients with cirrhosis: a meta-analysis. PLoS Med 2014;11:e1001624. 24691105 10.1371/journal.pmed.1001624 PMC3972088

[34] Nathani P , Gopal P, Rich N, Yopp A, Yokoo T, John B, . Hepatocellular carcinoma tumour volume doubling time: a systematic review and meta-analysis. Gut 2021;70:401–7. 32398224 10.1136/gutjnl-2020-321040 PMC7657990

[35] Rich NE , John BV, Parikh ND, Rowe I, Mehta N, Khatri G, . Hepatocellular carcinoma demonstrates heterogeneous growth patterns in a multicenter cohort of patients with cirrhosis. Hepatology 2020;72:1654–65. 32017165 10.1002/hep.31159 PMC7398837

[36] Asrani SK , Ghabril MS, Kuo A, Merriman RB, Morgan T, Parikh ND, . Quality measures in HCC care by the practice metrics committee of the American Association for the Study of Liver Diseases. Hepatology 2022;75:1289–99. 34778999 10.1002/hep.32240

[37] Singal AG , Lok AS, Feng Z, Kanwal F, Parikh ND. Conceptual model for the hepatocellular carcinoma screening continuum: current status and research agenda. Clin Gastroenterol Hepatol 2022;20:9–18. 32961340 10.1016/j.cgh.2020.09.036 PMC8287785

[38] Marquardt P , Liu PH, Immergluck J, Olivares J, Arroyo A, Rich NE, . Hepatocellular carcinoma screening process failures in patients with cirrhosis. Hepatol Commun 2021;5:1481–9. 34510836 10.1002/hep4.1735 PMC8435280

[39] Parikh ND , Tayob N, Al-Jarrah T, Kramer J, Melcher J, Smith D, . Barriers to surveillance for hepatocellular carcinoma in a multicenter cohort. JAMA Netw Open 2022;5:e2223504. 35867057 10.1001/jamanetworkopen.2022.23504 PMC9308050

[40] Walker M , El-Serag HB, Sada Y, Mittal S, Ying J, Duan Z, . Cirrhosis is under-recognised in patients subsequently diagnosed with hepatocellular cancer. Aliment Pharmacol Ther 2016;43:621–30. 26784271 10.1111/apt.13505 PMC4742403

[41] Marmot MG , Altman DG, Cameron DA, Dewar JA, Thompson SG, Wilcox M. The benefits and harms of breast cancer screening: an independent review. Br J Cancer 2013;108:2205–40. 23744281 10.1038/bjc.2013.177 PMC3693450

[42] Lin JS , Perdue LA, Henrikson NB, Bean SI, Blasi PR. Screening for colorectal cancer: updated evidence report and systematic review for the US preventive services task force. JAMA 2021;325:1978–98. 34003220 10.1001/jama.2021.4417 PMC13509038

[43] Huang DQ , El-Serag HB, Loomba R. Global epidemiology of NAFLD-related HCC: trends, predictions, risk factors and prevention. Nat Rev Gastroenterol Hepatol 2021;18:223–38. 33349658 10.1038/s41575-020-00381-6 PMC8016738

[44] Parikh ND , Tayob N, Singal AG. Blood-based biomarkers for hepatocellular carcinoma screening: approaching the end of the ultrasound era? J Hepatol 2023;78:207–16. 36089157 10.1016/j.jhep.2022.08.036 PMC10229257

[45] Park HJ , Kim SY, Singal AG, Lee SJ, Won HJ, Byun JH, . Abbreviated magnetic resonance imaging vs ultrasound for surveillance of hepatocellular carcinoma in high-risk patients. Liver Int 2022;42:2080–92. 34817921 10.1111/liv.15110

[46] Rich NE , Parikh ND, Singal AG. Overdiagnosis: an understudied issue in hepatocellular carcinoma surveillance. Semin Liver Dis 2017;37:296–304. 29272892 10.1055/s-0037-1608775 PMC8136685

[47] Atiq O , Tiro J, Yopp AC, Muffler A, Marrero JA, Parikh ND, . An assessment of benefits and harms of hepatocellular carcinoma surveillance in patients with cirrhosis. Hepatology 2017;65:1196–205. 27775821 10.1002/hep.28895 PMC5659110

[48] Singal AG , Patibandla S, Obi J, Fullington H, Parikh ND, Yopp AC, . Benefits and harms of hepatocellular carcinoma surveillance in a prospective cohort of patients with cirrhosis. Clin Gastroenterol Hepatol 2021;19:1925–32. 32920214 10.1016/j.cgh.2020.09.014 PMC7943645

