# Supplementary material for: Prediagnostic CT or MRI Utilization and Outcomes in Hepatocellular Carcinoma: SEER-Medicare Database Analysis
Source: Cancer Res Commun. 2023 May 16;3(5):874–83. doi: 10.1158/2767-9764.CRC-23-0075 (PMC10187587; doi:10.1158/2767-9764.CRC-23-0075)
Supplement: Supplementary Table S1 — shows the demographic and clinical factors associated with receipt of MRI/CT test in HCC patients. [file crc-23-0075-s01.docx]

**Supplementary Table S1. Factor associated with receipt of MRI/CT test.**

|  | **Univariate analysis** | | **Multivariable analysis** | |
| --- | --- | --- | --- | --- |
| **Characteristics** | **OR (95% CI)** | ***P* value** | **aOR (95% CI)** | ***P* value** |
| **Female Sex (Ref. Male)** | 1.22 (1.08-1.37) | 0.001 | 1.27 (1.12-1.44) | <0.001 |
| **Age** | 0.96 (0.95-0.97) | <0.001 | 0.97 (0.96-0.98) | <0.001 |
| **Race/ethnicity** | - | - | - | - |
| Non-Hispanic White | Ref | Ref | Ref | Ref |
| Non-Hispanic Black | 0.91 (0.74-1.13) | 0.39 | 0.75 (0.59-0.94) | 0.01 |
| Non-Hispanic API/Others | 1.10 (0.94-1.29) | 0.22 | 0.99 (0.83-1.18) | 0.93 |
| Hispanic | 1.25 (1.06-1.48) | 0.008 | 1.05 (0.88-1.26) | 0.57 |
| **Census Poverty Level** | - | - | - | - |
| <5% | Ref | Ref | Ref | Ref |
| 5% to <10% | 0.93 (0.79-1.10) | 0.43 | 0.93 (0.78-1.11) | 0.43 |
| 10% to <20% | 0.83 (0.71-0.98) | 0.02 | 0.80 (0.68-0.95) | 0.009 |
| 20% to 100% | 0.89 (0.76-1.05) | 0.17 | 0.84 (0.70-1.01) | 0.06 |
| **Rural-Urban** | - | - | - | - |
| Metro > 1 million | Ref | Ref | Ref | Ref |
| Metro 250k to 1 million | 0.92 (0.80-1.06) | 0.27 | 0.97 (0.83-1.12) | 0.66 |
| Metro < 250k | 1.03 (0.84-1.26) | 0.80 | 1.14 (0.92-1.41) | 0.25 |
| Non-Metro/Rural | 0.79 (0.67-0.95) | 0.01 | 0.92 (0.76-1.11) | 0.36 |
| **NCI comorbidity index** | - | - | - | - |
| Low (0 to 2) | Ref | Ref | Ref | Ref |
| Moderate (>2 to 4) | 1.05 (0.90-1.24) | 0.52 | 1.12 (0.95-1.32) | 0.19 |
| High (>4) | 1.01 (0.84-1.21) | 0.92 | 1.09 (0.90-1.32) | 0.40 |
| **Etiology** | - | - | - | - |
| HCV | Ref | Ref | Ref | Ref |
| NAFLD | 0.46 (0.40-0.53) | <0.001 | 0.52 (0.44-0.61) | <0.001 |
| ALD | 0.76 (0.65-0.89) | 0.001 | 0.74 (0.63-0.88) | 0.001 |
| HBV | 0.64 (0.49-0.84) | 0.001 | 0.67 (0.50-0.89) | 0.005 |
| Others/None | 0.48 (0.39-0.60) | <0.001 | 0.63 (0.50-0.80) | <0.001 |
| **Diabetes** | 1.20 (1.07-1.35) | 0.002 | 1.30 (1.14-1.48) | <0.001 |
| **Cirrhosis** | 1.88 (1.64-2.15) | <0.001 | 1.51 (1.28-1.78) | <0.001 |
| **Ascites** | 1.14 (1.02-1.27) | 0.02 | 0.78 (0.68-0.90) | <0.001 |
| **Hepatic encephalopathy** | 1.68 (1.45-1.95) | <0.001 | 1.34 (1.15-1.58) | <0.001 |

aOR, adjusted odds ratio; ALD, alcoholic liver disease; API, Asian/Pacific Islander; CT, computed tomography; HBV, hepatitis B virus; HCC, hepatocellular carcinoma; HCV, hepatitis C virus; OR, odds ratio; Metro, metropolitan; MRI, magnetic resonance imaging; NAFLD, nonalcoholic fatty liver disease; NCI, National Cancer Institute.
