# Supplementary material for: Prediagnostic CT or MRI Utilization and Outcomes in Hepatocellular Carcinoma: SEER-Medicare Database Analysis
Source: Cancer Res Commun. 2023 May 16;3(5):874–83. doi: 10.1158/2767-9764.CRC-23-0075 (PMC10187587; doi:10.1158/2767-9764.CRC-23-0075)
Supplement: Supplementary Table S2 — shows the demographic and clinical factors associated with overall survival for HCC patients after adjusting for lead-time bias. [file crc-23-0075-s02.docx]

**Supplementary Table S2. Factor associated with overall survival for HCC patients after adjusting for lead-time bias with mean sojourn time of 6 months.**

|  | **Univariate analysis** | | **Multivariable analysis** | |
| --- | --- | --- | --- | --- |
| **Characteristics** | **HR (95% CI)** | ***P* value** | **aHR (95% CI)** | ***P* value** |
| **Female Sex (Ref. Male)** | 0.86 (0.80-0.92) | <0.001 | 0.92 (0.86-0.99) | 0.03 |
| **Age** | 1.03 (1.02-1.03) | <0.001 | 1.02 (1.01-1.02) | <0.001 |
| **Race/ethnicity** | - | - | - | - |
| Non-Hispanic White | Ref | Ref | Ref | Ref |
| Non-Hispanic Black | 1.01 (0.89-1.15) | 0.86 | 1.00 (0.88-1.15) | 0.96 |
| Non-Hispanic API/Others | 0.65 (0.59-0.72) | <0.001 | 0.82 (0.74-0.92) | <0.001 |
| Hispanic | 0.94 (0.85-1.04) | 0.22 | 0.88 (0.79-0.97) | 0.01 |
| **Census Poverty Level** | - | - | - | - |
| <5% | Ref | Ref | Ref | Ref |
| 5% to <10% | 1.14 (1.03-1.26) | 0.01 | 1.06 (0.96-1.18) | 0.23 |
| 10% to <20% | 1.12 (1.02-1.24) | 0.02 | 1.05 (0.95-1.16) | 0.32 |
| 20% to 100% | 1.14 (1.03-1.26) | 0.009 | 1.11 (1.00-1.24) | 0.047 |
| **Rural-Urban** | - | - | - | - |
| Metro > 1 million | Ref | Ref | Ref | Ref |
| Metro 250k to 1 million | 1.15 (1.06-1.25) | 0.001 | 1.17 (1.07-1.27) | <0.001 |
| Metro < 250k | 1.23 (1.09-1.39) | 0.001 | 1.06 (0.93-1.20) | 0.40 |
| Non-Metro/Rural | 1.28 (1.15-1.42) | <0.001 | 1.10 (0.99-1.23) | 0.08 |
| **NCI comorbidity index** | - | - | - | - |
| Low (0 to 2) | Ref | Ref | Ref | Ref |
| Moderate (>2 to 4) | 1.23 (1.11-1.35) | <0.001 | 1.08 (0.98-1.19) | 0.11 |
| High (>4) | 1.60 (1.43-1.78) | <0.001 | 1.29 (1.15-1.44) | <0.001 |
| **Etiology** | - | - | - | - |
| HCV | Ref | Ref | Ref | Ref |
| NAFLD | 1.37 (1.27-1.49) | <0.001 | 1.22 (1.11-1.34) | <0.001 |
| ALD | 1.36 (1.24-1.50) | <0.001 | 1.10 (1.00-1.22) | 0.06 |
| HBV | 0.87 (0.73-1.03) | 0.10 | 1.07 (0.90-1.27) | 0.47 |
| Others/None | 1.21 (1.06-1.38) | 0.004 | 1.10 (0.96-1.26) | 0.18 |
| **Diabetes** | 1.10 (1.02-1.18) | 0.01 | 1.00 (0.92-1.08) | 0.98 |
| **Cirrhosis** | 1.06 (0.98-1.14) | 0.16 | 0.98 (0.89-1.08) | 0.74 |
| **Ascites** | 1.78 (1.67-1.91) | <0.001 | 1.85 (1.70-2.00) | <0.001 |
| **Hepatic encephalopathy** | 1.54 (1.41-1.68) | <0.001 | 1.46 (1.33-1.60) | <0.001 |
| **Imaging Type** | - | - | - | - |
| No imaging | Ref | Ref | Ref | Ref |
| US | 0.83 (0.76-0.91) | <0.001 | 1.00 (0.91-1.10) | 0.952 |
| CT/MRI | 0.66 (0.61-0.71) | <0.001 | 0.80 (0.74-0.87) | <0.001 |
| **Early-stage HCC**^a^ | 0.46 (0.42-0.50) | <0.001 | 0.56 (0.51-0.62) | <0.001 |
| **Curative treatment** | 0.27 (0.25-0.30) | <0.001 | 0.32 (0.29-0.35) | <0.001 |

^a^Single lesion ≤5 cm without vascular invasion or metastasis.

aHR, adjusted hazard ratio; ALD, alcoholic liver disease; API, Asian/Pacific Islander; CT, computed tomography; HBV, hepatitis B virus; HCC, hepatocellular carcinoma; HCV, hepatitis C virus; HR, hazard ratio; Metro, metropolitan; MRI, magnetic resonance imaging; NAFLD, nonalcoholic fatty liver disease; NCI, National Cancer Institute; US, ultrasound.
